# Determinants of Public-Private Partnership Adoption in Solid Waste Management in Rural China

**DOI:** 10.3390/ijerph17155350

**Published:** 2020-07-24

**Authors:** Dan Pan, Huan Chen, Guzhen Zhou, Fanbin Kong

**Affiliations:** 1 School of Economics, Jiangxi University of Finance and Economics, Nanchang 330013, China; pandan@jxufe.edu.cn (D.P.); chenhuan_0914@163.com (H.C.); zhouguzhen@jxufe.edu.cn (G.Z.); 2 School of Economics and Management, Zhejiang A&F University, Hangzhou 311300, China; 3 Research Academy for Rural Revitalization of Zhejiang Province, Zhejiang A&F University, Hangzhou 311300, China

**Keywords:** Public-Private Partnership (PPP), solid waste management, PPP adoption, China

## Abstract

Rural solid waste management is a severe challenge in China. The Public-Private Partnership (PPP) is an effective method for rural solid waste management. However, policy efforts aimed at stimulating the adoption of PPP in rural solid waste management have been limited in their success. This study aims to empirically investigate the determinants of rural solid waste management PPP adoption in China. First, this study builds a theoretical model that consists of factors related to the institutional environment and market and proposes theoretical hypotheses. Then, using the balanced provincial panel data of 150 samples from 2015 to 2019, this study applies various count regression models and truncated regression models to empirically test the theoretical hypotheses. The results show that provinces with higher fiscal transparency, financial burdens, and market demand tend to adopt more PPP, while provinces with lower per capita GDP and market openness index ratings have a stronger motivation to initiate more PPP. In contrast, investment institutional environment factors have no impact on PPP adoption. To stimulate the development of PPP in rural solid waste management, this study proposed that a good-governed government and a strong market demand are critical foundations, and also a debt-risk prevention and evaluation system should be established to avoid local debt risks resulting from over-adoption of PPP.

## 1. Introduction

Solid waste management is one of the greatest worldwide challenges, especially in China because of its large rural population and low economic development level [1,2]. It is reported that 300 million tons of solid waste is generated each year in rural China, and nearly 60% of them are discarded directly to the land or the water, causing profound adverse impacts on environmental protection, economic development, and human health [3,4].

As a basic need and public good, solid waste management used to be provided only by the local governments in China [5,6]. However, due to the financial constraints and the growing solid waste generation, merely depending on the local governments in solid waste management has resulted in a supply shortage of solid waste management facilities [7]. It is estimated that almost 40% of rural villages do not have adequate solid waste treatment facilities [3]. The Public-Private Partnership (PPP) has been regarded as an effective method to accelerate the supply of public goods and alleviate the government’s financial burden in that a PPP enables the government and private sectors to build a long-term cooperative relationship in public service supply [8,9].

To stimulate the development of PPPs, the Chinese central government has initiated a series of policies. For example, the “General Plan for the Reform of the Ecological Civilization System” [10], the “Guiding Opinions on Promoting Cooperation between Government and Social Capital in Agriculture” [11], and the “National Thirteenth Five-Year Plan for Comprehensive Rural Environment Improvement” [12] all stated clearly that local governments should adopt PPP in rural solid waste management. Some provinces, such as Fujian located in eastern China and Yunnan located in western China stipulated that PPPs are the only method that can be used to treat rural solid waste.

However, despite these massive initiatives to promote PPPs, the development of PPP in rural solid waste management has been relatively slow. According to the data from the Ministry of Finance, at the end of 2019, there were 12,341 total PPP projects with a total investment of 17.58 trillion RMB (or 2.49 trillion dollars), while the number of rural solid waste management PPP projects was only 190 with an investment of 52.2 billion RMB (or 7.4 billion dollars). Most provinces had less than ten rural solid waste management PPP projects, and six provinces even had zero rural solid waste management PPP projects. So, why have rural solid waste management PPP projects developed so slowly in the past few years? What are the main impediments that hampered the development of PPPs in rural solid waste management? How can we enhance the adoption of PPPs in rural solid waste management in China? These are the questions this study wants to answer. 

To answer the above questions, first, this study builds a theoretical model that consists of factors related to institutional environment and market and proposes theoretical hypotheses. Then, using the balanced provincial panel data of 150 samples from 2015 to 2019, this study applies various count regression models and truncated regression models to empirically test the theoretical hypotheses. The answers to the above questions are an essential prerequisite to the sustainable solid waste management in rural China and can provide insights into PPP development in China as well as in other developing countries with low economic development level and growing solid waste generation.

This study aims to make two principal contributions to the current literature. First, most existing research on the determinants of PPP adoption is based on the data from developed countries (see Wang et al. [13] for a literature review). There are limited systematic empirical studies that have been conducted in developing countries. Estimating the determinants of PPP adoption in developing countries is important because developing countries face higher financial pressure and increasing waste generation. Note that since China is the world’s biggest developing country and has the largest PPP database worldwide [14], investigating the factors affecting the adoption of rural solid waste management PPP projects in China could provide new insights into this question. Second, most literature about the PPP in China focused on the influencing factors of PPP adoption in total, while very little attention has been paid to the PPP adoption in rural solid waste management. This study offered a first-ever quantitative analysis of the determinants of PPP adoption in rural solid waste management.

The rest of the paper is laid out as follows. Section 2 provides a theoretical model and proposes hypotheses to demonstrate the effects of the institutional environment and market on the adoption of rural solid waste management PPP projects. Section 3 provides detailed information on the data, variable, and model. Section 4 reports the estimation results and presents some robustness checks and heterogeneous effects results. The final section gives conclusions.

## 2. Theoretical Framework and Hypotheses

**Figure 1:**
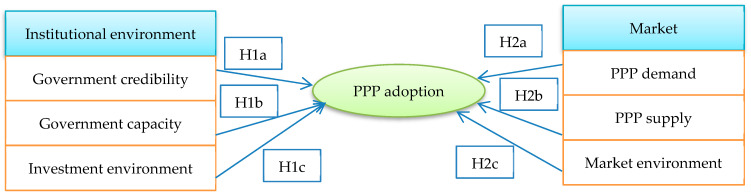
Theoretical framework and hypotheses used in this paper.

This study employed the theoretical framework developed by Yang and Hou [15] to investigate the critical determinates of PPP adoption in rural solid waste management. Based on the insights from previous studies, Yang and Hou [15] concluded that the institutional environment and the market factors are two primary drivers for PPP adoption. Figure 1 further elaborates on this framework and proposes the hypotheses.

### 2.1. Institutional Environment

The institutional environment factor reflects the essential elements that constituting a sound PPP conducive environment [15]. Private enterprises may face substantial uncertainties when being involved in a PPP. A suitable institutional environment can help to reduce conflicts among different stakeholders, decrease opportunistic behaviors, and increase the profit of a PPP, and thus facilitate the involvement of private sectors and PPP adoption [16]. Brewer and Hayllar [17] found that a sound institutional environment is beneficial to the long-term cooperation between the private sectors and the government, which plays a critical role in PPP initiation. Based on a qualitative comparative analysis, Soecipto and Verhoest [18] concluded that a well-developed institutional environment could bolster PPP development. The main elements that constitute the institutional environment include the government institutional environment and investment institutional environment.

The successful development of PPP depends on a capable, credible, and skilled government, especially in a transition economy country like China [16]. Kort and Klijn [19] pointed out that the management strategy of the government rather than the organizational feature is a determinative factor in fostering PPP development. Based on a systematic review of 122 PPP cases in 27 countries, Xiong et al. [20] pointed out that a good governance practice is critical for PPP success. Government credibility and capacity are two key factors that compose the government institutional environment.

Government credibility shows the degree of responsibility and responsiveness of the government in all contracts, which is a prerequisite for attracting private enterprises to invest in PPPs [21]. The higher the government credibility, the lower the risk of over-investment and information asymmetry, and therefore the higher willingness of the private sectors to invest in PPP. Also, when cooperating with a credible government, private enterprises can have a clear picture of the government’s decision and thereby can avoid the opportunistic behaviors of the government, which will reduce the collaboration costs and foster the development of PPPs [22]. Rosell and Saz-Carranza [23] concluded that government credibility effectively contributes to PPP implementation by analyzing the PPP development in 135 countries worldwide.

Government capacity, especially fiscal capacity, is essential for successful PPP adoption. The PPP is regarded as a useful tool to alleviate the financial burden and mitigate the debt risk of local governments [24]. As such, governments with a lower fiscal capacity or higher financial burden will be more likely to adopt a PPP to promote rural solid waste management infrastructure construction and operation. Based on a systematic literature review of 186 articles, Wang et al. [13] concluded that fiscal capacity is a leading factor that contributes to the adoption of PPP.

In addition, a favorable investment institutional environment can also promote PPP adoption by encouraging private enterprises to invest in PPP.

Hence, this study proposes the first hypothesis:

**Hypothesis** **1a.** *Higher government credibility is positively associated with PPP adoption.*

**Hypothesis** **1b.** *Lower government capacity is positively associated with PPP adoption.*

**Hypothesis** **1c.** *A favorable investment institutional environment leads to faster PPP adoption.*

### 2.2. Market

The market factor is a prerequisite for PPP implementation, since it determines the profit that private investors can get from PPP [16]. A stable and flourishing market is a triggering incentive for private enterprises to participate in PPP investment for that pursuing profit is the primary consideration of private enterprises. A key factor that impacts profitability is the gap between the demand and supply for rural solid waste management infrastructure construction. An economy with higher rural solid waste management infrastructure demand and a lower rural solid waste management infrastructure supply will bring about higher profit for PPP implementation [25]. Meanwhile, a well-developed market environment can facilitate PPP adoption by reducing transaction costs between governments and private enterprises.

Thus, this study proposes the second hypothesis:

**Hypothesis** **2a.** *Governments with a higher rural solid waste management infrastructure demand tend to have a stronger motivation to adopt PPPs.*

**Hypothesis** **2b.** *Governments with a lower rural solid waste management infrastructure supply are more likely to adopt PPPs.*

**Hypothesis** **2c.** *A well-developed market environment is beneficial to PPP adoption.*

## 3. Data and Method

### 3.1. Data Source

The analysis of this paper was based on a balanced panel data in 30 Chinese provinces from 2015 to 2019. Due to missing data, Tibet is not included. The total sample size of the balanced panel dataset was 150, which equaled 30 provinces multiplied by five years (from 2015 to 2019). The reason for choosing the study period from 2015 to 2019 is that there were no rural solid waste management PPP projects before 2015.

Specifically, this study manually searched with the keyword “rural solid waste” in the PPP project database [26] in May 2020 to collect the data of rural solid waste management PPP projects. The PPP project database was developed by the Ministry of Finance in China in 2013 and has now become the largest PPP database worldwide. This database covered all PPP projects, including transportation, education and culture, health and social security, tourism, ecological construction, and water supply, among other activities [16]. The data of the independent variables influencing rural solid waste management PPP adoption mainly comes from China Statistic Yearbooks.

### 3.2. Dependent Variables

The dependent variable of this paper is rural solid waste management PPP adoption. Two indicators are used to measure it: the number and investment of rural solid waste management PPP projects initiated by each province in each year.

**Figure 2:**
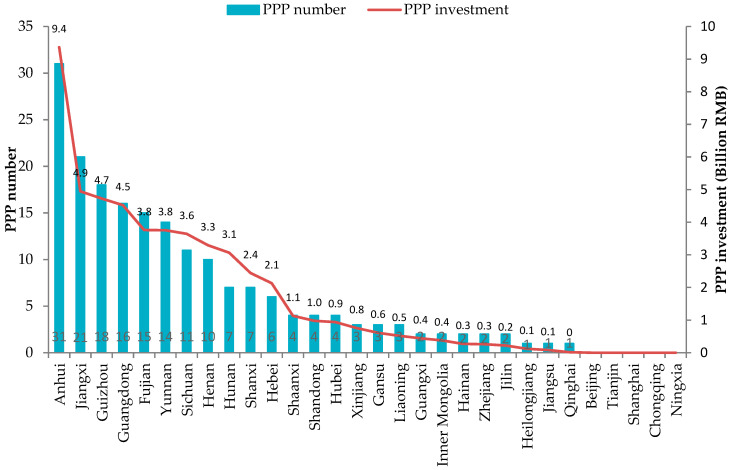
The spatial distribution of the total number and investment of rural solid waste management Public-Private Partnership (PPP) projects over five years from 2015 to 2019.

From 2015 to 2019, there were a total of 190 rural solid waste management PPP projects with a total investment of 52.2 billion RMB (about 7.4 billion dollars). Figure 2 shows the spatial distribution of the total number and investment of rural solid waste management PPP projects over five years from 2015 to 2019 in each province. It can be seen that there are significant spatial disparities in rural solid waste management PPP adoption. The total number and investment of rural solid waste management PPP projects are higher in less developed provinces located in Central and Western China. For example, Anhui, Guizhou, and Shanxi provinces—three middle-income provinces in China—ranked the top three in the total number of rural solid waste management PPP. However, some economically-developed provinces like Zhejiang and Jiangsu provinces had fewer PPP projects, and no PPP projects were even found in Beijing and Shanghai—two of the wealthiest areas in China.

### 3.3. Independent Variables

#### 3.3.1. Institutional Environment

Following Tan and Zhao [16], this study took fiscal transparency (Ftran) as a measurement of government credibility. In a transition country like China, where the financial market is not mature, fiscal transparency is the most crucial aspect of government credibility to attract private enterprises to involve themselves in PPPs. This study obtained the data of fiscal transparency from China’s Fiscal Transparency Report released by the Shanghai University of Finance and Economics.

Following Wang et al. [24], this study used the financial burden (Fburden) to represent the government capacity. A higher financial burden, which expresses higher financial pressure on the government, could encourage governments to adopt more PPP. There are various measurements of financial burden, including the absolute indicators such as financial revenue, financial expenditure, and the difference between fiscal expenditure and fiscal revenue [27], and the relative indicators such as the ratio of fiscal revenue to fiscal expenditure [28], and the difference between fiscal expenditure and fiscal revenue divided by gross domestic product (GDP). In comparison, the relative indicators can better reflect the degree of the financial burden and avoid the problem of incommensurability resulting from the absolute indicators. Thus, this study used the difference between fiscal expenditure and fiscal revenue divided by GDP to measure financial burden. This data comes from China Statistic Yearbooks.

This study used the financial development index (Finance) and the ratio of fixed-asset investment to GDP (Finvest) to measure the investment institutional environment. A mature investment institutional environment is beneficial to PPP operation. Finance was measured by the ratio of the financial institutions’ loan balance to the fixed asset investment. The data of Finance comes from China Financial Statistics Yearbook, and the data of Finvest comes from China Statistic Yearbooks.

#### 3.3.2. Market

As stated in the theoretical framework section, PPP demand, PPP supply, and market environment are three main factors of the market. This study used the total population (LnP), per capita GDP (Pgdp), the growth of per capita GDP (Gpgdp), and urbanization rate (Urban) to represent PPP demand. These four indicators can reflect people’s real and potential need for rural solid waste management, which are positively associated with higher PPP adoption. This study used the treatment rate of rural solid waste in each province (Wtreat) to indicate PPP supply. The higher the rural solid waste treatment rate, the lower the possibilities to initiate more PPPs. The data of these indicators come from China Statistic Yearbooks.

Following Zhang et al. [25], this study used the market openness index (Market) to reflect the market environment. A higher market openness index means a lower monopoly or oligopoly share of the market owned by governments, which may, in turn, result in more participation of private sectors to rural solid waste management. This study obtained the data of market openness index from the Marketization Index of China’s Provinces released by the National Economic Research Institute [29].

Table 1 shows the definition and summary statistics of the variables. It should be noted that the independent variables have been lagged by one year, which is from 2014 to 2018, while the dependent variables are based on the data from 2015 to 2019. The reason for the use of lagged data is that government’s PPP adoption decisions are usually based on the previous year’s condition rather than the current year’s ones. This approach can also account for potential endogeneity problems resulting from two-way causality [24].

Table 2 reports the Pearson correlation matrix among the dependent variables and independent variables. According to the criteria of Miserendino and Pizzolon [30], variables with Pearson correlation coefficients higher than 0.65 may have high multicollinearity and should be removed before the regression. As Table 2 shows, all the Pearson correlation coefficients are below 0.65, indicating that all the independent variables can enter into the regression. Meanwhile, the correlation matrix also provides preliminary results of the determinates of rural solid waste management PPP adoption. PPPN and LNPPPI correlated positively and significantly with Ftran, Fburden, Gpgdp, Lnp, and Urban, and correlated negatively and significantly with Lnpgdp and Market. However, these exploratory results do not exclude the mixed effects of other variables. Therefore, there is a need to use econometric methods to quantitatively analyze the relationship between rural solid waste management PPP adoption and the independent variables.

### 3.4. Method

To investigate the determinants of PPP in rural solid waste management, this study employed the following Equation:

(1) $${\mathrm{PPP}}_{it}=\beta_{0}+\beta_{1}Inenvironment_{it}+\beta_{2}Market_{it}+\mu_{it}$$

where i represents the province and t represents the year; $PPP_{\mathrm{it}}$ denotes the number and the investment of rural solid waste management PPP projects in province i in year t; $Inenvironment_{it}$ denotes the institutional environment variables; $Market_{it}$ represents the market variables; $\mu_{it}$ is the error term.

The first dependent variable—the number of rural solid waste management PPP projects (PPPN), is a count variable that censored between 0 and 14, with 0 reflecting no rural solid waste management PPP projects and 14 indicating the maximum number of rural solid waste management PPP projects. Ordinary least squares (OLS) is not suitable for estimation count data. Therefore, this study employed two commonly used count data models—the Poisson regression model and the negative binomial (NB) model—to estimate Equation (1) [31]. The Poisson regression model is the most widely used approach that assumes the mean of PPPN is equal to the variance of PPPN, which is a strong assumption. In contrast, the NB model does not require this assumption and is more suitable for the existence of overdispersion in count data that when the mean does not equal the variance [32].

The second dependent variable—the investment of rural solid waste management PPP projects (LNPPPI)—is a censored variable, as some provinces have zero investment in rural solid waste management PPP. This study employed the Tobit model—a typical truncated regression method—to estimate the determinates of LNPPPI. Meanwhile, this study also used the generalized least square method (GLS) as a robustness check for the Tobit model.

## 4. Empirical Results and Discussions

### 4.1. Main Results

Table 3 reported the main findings of the determinates of PPP adoption. The Poisson and NB model can be estimated with both random-effects and fixed-effects models. Based on the Hausman test results, this study used the fixed-effects model. Columns 1 and 2 present the fixed-effects results for PPPN. The observations in the fixed-effects models of Columns 1 and 2 are 125. This is because there are five provinces—Beijing, Tianjin, Shanghai, Chongqing, and Ningxia, have zero rural solid waste management PPP, so data from these five provinces cannot enter into the fixed-effects estimation. It can be seen that the estimation results of the Poisson and NB model are almost the same, indicating the robustness of the results. However, this study illustrated the results based on the NB model for the following reasons. First, as shown in Table 1, the variance value of PPPN is much larger than its mean value, indicating the existence of overdispersion, which is not suitable for the Poisson model [33]; second, the P-value of the likelihood ratio test is statistically significant, which also indicates that the NB model is preferred to the Poisson model [34]. Columns 3 and 4 present the results of the random effect of the Tobit model and the GLS model for LNPPPI. This study explained the results based on the Tobit model.

In terms of the institutional environment variables, the results are mostly in line with the hypotheses. The government’s credibility as measured by fiscal transparency (Ftran) and the government capacity as measured by financial burden (Fburden) are both positively related to PPP numbers and PPP investment. This result indicates that the greater the fiscal transparency, the higher possibilities of PPP adoption, which is in agreement with previous studies of PPP [21,22,23]. Fiscal transparency can boost private enterprises’ trust in the government and lower the likelihood of the government’s opportunistic behaviors, and thus improves the probability of PPP implementation. The results also suggest that governments with higher financial burdens are more likely to adopt PPP. Similar findings were obtained by Wang et al. [13], who stated that the fiscal burden is a triggering incentive for PPP adoption. Building solid waste management PPP projects in rural China is costly. PPP is regarded as an effective method to partly mitigate the financial burden on the government. Therefore, PPP projects are more likely to be implemented in areas with a higher financial burden. However, the effects of the financial development index (Finance) and the ratio of fixed-asset investment to GDP (Finvest) were not significant, suggesting that investment environment variables are not the main determinants of PPP adoption decision. This finding was also reported by Yang and Hou [15].

Regarding the market variables, as expected, the coefficients of the three market demand variables—the growth rate of per capita GDP (Gpgdp), the total population (Lnp), and the urbanization rate (Urban), are all significantly positive, indicating that PPP projects are more possible to reach deals in areas with a higher growth rate of per capita GDP, more population, and a higher urbanization rate. This result is in accordance with the conclusion of previous literature [25]. However, unexpectedly, the effect of the market supply variable—the treatment rate of rural solid waste (Wtreat), is not significant, meaning that infrastructure supply shortage is not the main obstacle in fostering PPP. A similar finding was also obtained by Zhang et al. [25]. Besides, the coefficient of per capita GDP (Lnpgdp) is significantly negative, suggesting that the lower the per capita GDP, the higher the need for PPP projects. This result contradicts expectations. A possible explanation is that less-developed governments with lower per capita GDP have a less financial budget to invest in rural solid waste management infrastructure construction and operation, and thus tend to have a stronger motivation to adopt PPP to alleviate their fiscal burden.

However, the coefficient of market openness index (Market) is significantly negative, indicating that the higher the market openness index, the less the motivation for PPP adoption. This result is not in line with the expectations. The reason for this result may partly be explained by the fact that areas with a high market openness index mostly have high per capita GDP: the data shows that the correlation coefficient between market openness index and per capita GDP is 0.58. According to the negative relationship between per capita GDP (Lnpgdp) and PPP adoption, there is also a negative relationship between the market openness index (Market) and PPP adoption.

**Figure 3:**
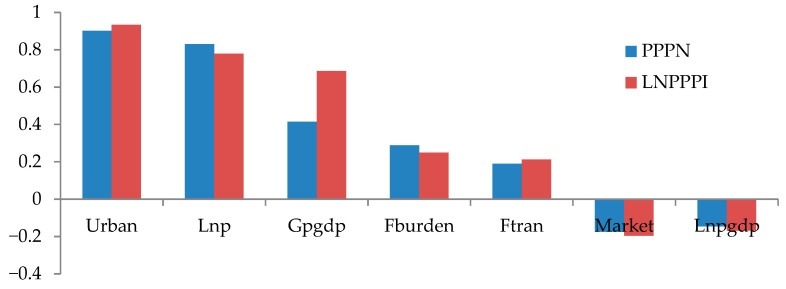
The relative importance of explanatory variables according to the standardized coefficients method.

In addition, in order to identify the relative effects of each explanatory variable on PPP adoption, this study used the “standardized coefficients” method to rank the explanatory variables. A comprehensive understanding of the relative importance of explanatory variables could contribute to a prioritization of potential public policies. The standardized coefficients are adjusted coefficients that can be directly compared. They denote the predicted change in the probability of PPP adoption associated with one standard deviation change in the explanatory variables [35]. Figure 3 shows the relative effects of each explanatory variable using the standardized coefficients method. The order of the importance was as follows: Urban, Lnp, Gpgdp, Fburden, Ftran, Market, and Lnpgdp.

### 4.2. Robustness Check

To check the robustness of the above results, four robustness checks were conducted.

First, a robustness regression was conducted by including only the samples with the PPP projects in the implementation and transfer stage. The reason for undertaking this robustness check is as follows. There are five stages in PPP projects: identification, preparation, procurement, implementation, and transfer. Among them, the first three stages can only indicate the government’s intention to adopt PPP, rather than their real adoption behavior of PPPs. Many PPP projects in the first three stages cannot reach deals or will be withdrawn when they come into the last two stages. Only when the PPP projects enter into the last two stages does this indicate a successful adoption [16]. This robustness regression result was presented in Columns 1 and 2 in Table 4. It can be found that the coefficients of most variables essentially remain unchanged, supporting the stability of the baseline regression results.

Second, considering the differences in the regional scale in China’s provinces, this study carried out a robustness test by replacing the total number and investment of rural solid waste management PPP projects with the per capita number and investment of rural solid waste management PPP projects. It can be seen that there is little difference between the results displayed in Columns 3 and 4 in Table 4 and the baseline results in Table 3, indicating that the baseline regression results are robust.

Third, this study used the ratio of fiscal revenue to fiscal expenditure as a substitute indicator of the financial burden to test the robustness. The results displayed in Columns 5 and 6 in Table 4 showed that there is a positive relationship between financial burden and PPP adoption, which is in line with the conclusion in Table 3, suggesting the robustness of the baseline regression results.

Fourth, due to the considerable variation in PPP among different provinces, data outliners in the samples may affect estimation results, so this study winsorized the up and bottom 1% of the samples to lessen the impact of extreme values. The results were presented in Columns 7 and 8 in Table 3. It can be seen that the results after winsorizing are almost the same as the baseline regression results, supporting the robustness of results.

### 4.3. Heterogeneous Effects across Different Regions

As shown in Figure 2 in Section 3.2, there are significant spatial disparities in rural solid waste management PPP adoption across different regions. A further asked question is whether the determinates of PPP adoption show regional heterogeneity or not. To answer this question, this study conducted a heterogeneity analysis using two sub-samples from Eastern China, and Central and Western China. The results were presented in Table 5. The results of PPPN were based on the NB model, and the results of LNPPPI were based on the Tobit model. 

The results in Table 5 showed that, for Eastern China, three market demand variables—the growth rate of per capita GDP (Gpgdp), the total population (Lnp), and the urbanization rate (Urban), had significant effects on PPP adoption, while other variables had no significant effects. For Central and Western China, three variables—fiscal transparency (Ftran), financial burden (Fburden), and market openness index (Market)—had significant effects on PPP adoption, while the above three market demand variables were not significant, which was the opposite of the results of Eastern China. These results indicated that the motivation of PPP adoption in Eastern China is more due to market demand factors, while the promotion of PPP projects in Central and Western China stems more from institutional environment factors.

## 5. Conclusions

PPP is an effective way in rural solid waste management by reducing the shortage of funds, technology, and personnel needed. However, policy efforts aimed at stimulating the adoption of PPP in rural solid waste management have been limited in their success. Based on a provincial panel data of 150 samples from 2015 to 2019, this paper employs various count regression models and truncated regression models to empirically examine the determinates of PPP in rural solid waste management. This study found that the government environment factors—fiscal transparency and financial burden, and market demand factors—the growth rate of per capita GDP, total population, and urbanization rate, are all positively related to PPP adoption. The per capita GDP and market openness index have a negative impact on PPP adoption. In contrast, the effects of the investment environment factors—the financial development index and the ratio of fixed-asset investment to GDP, and the market supply factor—the treatment rate of rural solid waste, are not significant.

Based on the above results, this study makes the following three practical policy implications for the effective implementation of rural solid waste management PPP projects for China and beyond.

First, it is important to increase fiscal transparency to promote government governance. The results show that a good-governed government is a critical prerequisite for PPP adoption. Therefore, local governments should appropriately increase the transparency of government decision-making information, eliminate hidden barriers to PPP projects construction, and attract the participation of more social capital. Only with good government governance can the PPP model be expected to become a new type of rural solid waste management.

Second, it is important to build up a debt-risk prevention and evaluation system to avoid local debt risks resulting from over-adoption of rural solid waste management PPP projects. The results show that provinces with a heavy financial burden tend to adopt rural solid waste management PPP projects. However, choosing PPPs does not mean that local governments can leave an issue alone. If the local government blindly promotes PPP projects without taking into account its financial resources, it will be challenging to support the continued operation of PPP projects in the future and thus will lead to further local debt risks and seriously hinder regional economic development. Therefore, for areas with a high financial burden, it is necessary to establish a local debt risk-based system. This would help to reduce the over-investment of PPPs and thus increase the efficiency of PPP development.

Third, local governments might match the PPP projects to the local economy to prevent the over-investment of PPP. The results show that poor provinces are more likely to adopt rural solid waste management PPP projects. Although PPP can reduce the governments’ total investment to a certain extent, many rural solid waste management PPP projects will still desire government investment in the future. Therefore, local governments should take into account their actual situation and make appropriate use of PPPs on the premise of considering their financial ability to pay, not merely as a tool to alleviate debt pressure and solve financing difficulties, but based on the fundamental purpose of improving the public welfare of rural people. This is especially important in Central and Western China, as the results of the heterogeneous effects show that market demand factors that reflect peoples’ demand are not the primary determinates of PPP adoption in Central and Western China.

Although this study adds to the literature by quantitatively examining the determinants of PPP adoption in rural solid waste management based on count regression models and truncated regression models, there are still some limitations. First, this study does not account for the spatial effects that may exist in PPP adoption. For example, rural solid waste is not only associated with a single region but also may be related to the other areas with less disposal capacity. Therefore, the implementation of rural solid waste management PPP projects does not only depend on the growth of the specific population of an area, but also on the other regions. Such spatial effects may be further examined through the spatial regression model. Second, while this study used the macro-level data from each province, further studies based on the micro-level data, such as questionnaire surveys, are necessary to reveal the underlying mechanisms of the determinates of rural solid waste management PPP adoption.

## Figures and Tables

**Table 1:** Descriptive statistics of variables.

Variables	Definition	Mean	SD	Min	Max
Dependent variables					
PPPN	The number of solid waste management PPP projects in each year from 2015 to 2019	1.267	2.260	0	14
LNPPPI	The investment of solid waste management PPP projects (in logs) in each year from 2015 to 2019	4.736	5.296	0	13.172
Institutional environment variables					
Ftran	The fiscal transparency index released by the Shanghai University of Finance and Economics	38.304	14.025	14.0	77.7
Fburden	The financial burden measured by the difference between fiscal expenditure and fiscal revenue divided by GDP	0.139	0.101	0.014	0.516
Finance	The financial development index measured by the ratio of the financial institutions’ loan balance to the fixed asset investment	2.040	1.809	0.856	9.271
Finvest	The ratio of fixed asset investment to GDP	0.845	0.275	0.233	1.480
Market variables					
Lnpgdp	Per capita GDP (RMB, in logs)	10.885	0.399	10.172	11.851
Gpgdp	The growth rate of per capita GDP	1.066	0.044	0.777	1.184
Lnp	Total population (10^4^ person, in logs)	8.211	0.734	6.368	9.337
Urban	Urbanization rate measured by the ratio of the urban population to the total population	58.762	11.491	40.010	89.600
Wtreat	The treatment rate of rural solid waste	47.108	28.249	1.6	98.0
Market	The market openness index released by the National Economic Research Institute	6.913	1.965	2.372	11.109

Notes: These terms are abbreviated for ease of read—PPPN (PPP number), LNPPPI (ln(PPP investment)), Ftran(fiscal transparency), Fburden(financial burden), Finance(financial development index), Finvest(fixed-asset investment to GDP), Lnpgdp(ln(per capita GDP)), Gpgdp(growth rate of per capita GDP), Lnp(ln(population)), Urban(Urbanization rate), Wtreat(treatment rate), Market(market openness index).

**Table 2:** Pearson correlation coefficients among the variables.

Variables	PPPN	LNPPPI	Ftran	Fburden	Finance	Finvest	Lnpgdp	Gpgdp	Lnp	Urban	Wtreat	Market
PPPN	1.000											
LNPPPI	0.700 ***	1.000										
Ftran	0.036 *	0.022 *	1.000									
Fburden	0.003 **	0.002 *	−0.013	1.000								
Finance	−0.193	−0.260	0.118	−0.118	1.000							
Finvest	0.198	0.204	−0.206	0.260	−0.527 **	1.000						
Lnpgdp	−0.285 ***	−0.298 ***	0.096	−0.120	0.301	−0.277	1.000					
Gpgdp	0.198 **	0.185 **	0.024	−0.099	0.101	−0.001	0.044	1.000				
Lnp	0.256 ***	0.376 ***	0.062	−0.175	−0.160	−0.211	−0.014	0.125	1.000			
Urban	0.270 ***	0.354 ***	0.094	−0.181	0.261 **	−0.569 **	0.220 *	−0.046	−0.129	1.000		
Wtreat	−0.095	−0.093	0.260	−0.251	0.494	−0.324	0.196	0.245 **	0.178	0.330 **	1.000	
Market	−0.106 *	−0.136 *	0.119	−0.136 **	0.485 **	−0.490	0.580 ***	0.134	0.192	0.245 **	0.253	1.000

Notes: *** *p* < 0.01 ** *p* < 0.05; * *p* < 0.1.

**Table 3:** The baseline estimation results of the determinates of PPP in rural solid waste management.

Variables	PPPN		LNPPPI	
	Poisson Model	NB Model	Tobit Model	GLS Model
Ftran	0.020 **(0.009)	0.022 *(0.012)	0.082 *(0.042)	−0.065 **(0.028)
Fburden	24.524 **(10.775)	16.952 *(8.842)	30.101 *(16.704)	84.946 **(34.972)
Finance	−0.198 (0.298)	−0.027 (0.353)	−0.268 (1.168)	0.016 (0.421)
Finvest	0.687 (1.067)	0.077 (1.269)	5.807 (6.256)	3.366 (2.860)
Lnpgdp	−3.174 ***(1.046)	−3.192 ***(0.968)	−12.116 *(6.389)	−12.425 **(5.911)
Gpgdp	9.354 **(4.042)	9.503 **(3.903)	42.877 *(23.760)	17.870 *(9.660)
Lnp	1.575 ***(0.387)	2.865 **(1.293)	8.538 ***(2.449)	3.662 ***(0.975)
Urban	0.358 **(0.172)	0.141 ***(0.049)	0.568 *(0.339)	0.281 *(0.148)
Wtreat	−0.017 (0.012)	−0.010 (0.013)	0.033 (0.050)	0.027 (0.023)
Market	−0.501 **(0.253)	−0.606 **(0.278)	−2.060 *(1.134)	−1.016 *(0.523)
Constant	1.954 (10.140)	−59.438 **(23.682)	−88.303 (66.311)	−19.250 (27.935)
Observations	125	125	150	150
Log-likelihood	−121.079	−108.627	−274.243	−391.818

Notes: Standard errors are reported in parentheses. *** *p* < 0.01; ** *p* < 0.05; * *p* < 0.1.

**Table 4:** The robustness checks of the baseline estimation results.

Variables	PPP Projects in the Implementation and Transfer Stage		Per Capita PPP Projects		Replacement of Financial Burden Indicator		Winsorizing Extreme Values	
	PPPN	LNPPPI	PPPN	LNPPPI	PPPN	LNPPPI	PPPN	LNPPPI
	NB Model	Tobit Model	NB Model	Tobit Model	NB Model	Tobit Model	NB Model	Tobit Model
Ftran	0.038 **	0.146 *	0.026 *	0.121 **	0.022 *	0.079 *	0.022 *	0.094 *
	(0.016)	(0.086)	(0.014)	(0.052)	(0.012)	(0.042)	(0.012)	(0.051)
Fburden	28.549 *	32.296 *	2.742 **	2.755 *	1.543 **	4.823 **	21.055 *	85.277 *
	(14.827)	(17.250)	(1.044)	(1.650)	(0.768)	(2.176)	(11.719)	(49.703)
Finance	−0.718	−0.234	−0.214	0.019	0.024	−0.237	0.228	0.174
	(0.971)	(1.770)	(9.101)	(0.308)	(0.332)	(1.129)	(0.458)	(0.558)
Finvest	−0.478	4.000	2.053	0.989	−0.017	6.045	0.792	5.371
	(2.047)	(2.657)	(37.667)	(1.311)	(1.266)	(6.230)	(1.679)	(3.468)
Lnpgdp	−2.599 **	−1.767 **	−3.402 **	−2.952 **	−3.123 ***	−2.398 **	−2.754 **	14.456 *
	(1.269)	(0.704)	(1.656)	(1.255)	(1.023)	(1.181)	(1.148)	(7.626)
Gpgdp	11.114 *	69.067 **	14.042 *	8.041 *	9.651 **	42.223 *	7.679 **	10.635 **
	(6.224)	(33.116)	(8.122)	(4.833)	(3.878)	(23.846)	(3.662)	(4.406)
Lnp	3.400 **	10.165 ***	0.269 **	0.117 **	2.127 *	8.377 ***	3.177 *	4.260 ***
	(1.703)	(3.264)	(0.126)	(0.048)	(1.101)	(2.236)	(1.679)	(1.130)
Urban	0.179 ***	0.272 **	0.117 **	0.091 *	0.433 **	0.717 **	0.119 **	0.103 *
	(0.061)	(0.414)	(0.054)	(0.048)	(0.168)	(0.347)	(0.053)	(0.055)
Wtreat	−0.014	0.061	0.003	−0.002	−0.008	0.033	−0.007	0.023
	(0.018)	(0.066)	(0.303)	(0.010)	(0.013)	(0.050)	(0.013)	(0.025)
Market	−0.869 **	−4.771 ***	−0.242 *	−0.185 *	−0.583 **	−2.098 *	−0.609 *	−0.916 *
	(0.397)	(1.568)	(0.133)	(0.103)	(0.276)	(1.087)	(0.319)	(0.487)
Constant	−67.870 **	−144.079	4.718	10.236	−46.820 **	−85.196	−70.077 **	−34.606
	(31.288)	(89.070)	(407.732)	(13.975)	(23.423)	(65.233)	(30.651)	(33.926)
Obs.	115	150	150	150	150	150	133	133

Notes: Standard errors are reported in parentheses. *** *p* < 0.01; ** *p* < 0.05; * *p* < 0.1.

**Table 5:** The heterogeneous effects results of the determinates of PPP in rural solid waste management across different regions.

Variables	PPPN	LNPPPI	PPPN	LNPPPI
	Eastern China	Central and Western China	Eastern China	Central and Western China
Ftran	0.008 (0.017)	0.028 **(0.012)	0.044 (0.149)	0.024 *(0.013)
Fburden	0.595 (12.842)	14.805 ***(5.793)	50.528 (111.464)	65.237 ***(23.027)
Finance	−0.329 (0.401)	−0.349 (0.559)	−0.391 (2.829)	0.639 (2.895)
Finvest	−2.071 (2.934)	1.102 (1.256)	−2.451 (24.827)	7.455 (6.654)
Lnpgdp	−2.179 (3.213)	−1.157 (1.736)	5.733 (27.134)	1.241 (6.926)
Gpgdp	27.339 **(12.601)	6.928 (4.570)	256.989 **(108.010)	16.085 (22.785)
Lnp	2.596 ***(0.890)	0.678 (0.535)	13.818 ***(3.926)	7.586 (5.367)
Urban	0.144 *(0.087)	−0.004 (0.101)	0.347 ***(0.103)	−0.489 (0.904)
Wtreat	−0.015 (0.022)	0.009 (0.009)	−0.274 (0.179)	0.076 (0.051)
Market	0.080(0.453)	−0.597 ***(0.229)	0.827 (3.399)	−3.345 ***(1.257)
Constant	−7.998 (33.502)	−21.613 (17.577)	−361.707 (285.057)	−151.649 *(78.615)
Observations	55	95	55	95
Log-likelihood	−50.352	−140.327	−76.249	−201.412

Notes: Standard errors are reported in parentheses. *** *p* < 0.01; ** *p* < 0.05; * *p* < 0.1.
